# Inversely Correlated Restoration of Body Condition Score and Systemic Metabolic Burden in Lactating Cows: Implications for Milk Fat Globule Size and Mitigation of Negative Energy Balance Effects on Milk Fat Composition

**DOI:** 10.3390/ijms26094296

**Published:** 2025-05-01

**Authors:** Krzysztof Młynek, Kalina Wnorowska, Agata Danielewicz, Antonio Natalello, Kamila Puppel

**Affiliations:** 1 Institute of Animal Science and Fisheries, University of Siedlce, ul. B. Prusa 14, 08-110 Siedlce, Poland; krzysztof.mlynek@uws.edu.pl (K.M.); kw88137@stud.uws.edu.pl (K.W.); agata.danielewicz@uws.edu.pl (A.D.); 2 Department of Agriculture, Food and Environment, University of Catania, 95123 Catania, Italy; antonio.natalello@unict.it; 3 Departments of Animal Breeding, Institute of Animal Sciences, Warsaw University of Life Sciences, Ciszewskiego 8, 02-786 Warsaw, Poland

**Keywords:** cattle, energy balance, metabolism, NEB markers, milk fat globules

## Abstract

In lactating dairy cows, negative energy balance (NEB) induces metabolic shifts, including enhanced lipolysis, leading to elevated concentrations of free fatty acids (FFAs) in circulation. Metabolic changes affect milk fat synthesis and the characteristics of milk fat globules (MFGs), particularly their size and distribution. Systemic FFA release inversely correlates with the restoration of the body condition score (BCS), suggesting that recovering the BCS may mitigate the negative effects of NEB on milk fat composition. This study aimed to investigate the relationship between BCS restoration, metabolic burden, and their effects on MFG characteristics in lactating cows. The study was conducted on two dairy farms (F1 and F2) with 80 Holstein-Friesian cows. Cows were grouped according to farm and diet, with average lactation yields of 9653 ± 259 kg (F1) and 9548 ± 341 kg (F2). Milk composition was analyzed, and blood and milk samples were collected at four lactation stages. The results showed a significant correlation between elevated circulating FFA concentrations, resulting from adipose tissue lipolysis during NEB, and alterations in MFG size and distribution. The restoration of BCS was inversely correlated with FFA release, suggesting that improvements in the BCS may mitigate the adverse effects of NEB on milk fat synthesis by regulating lipolysis. Additionally, higher β-hydroxybutyrate (BHBA) levels were associated with a reduction in MFG diameter, indicating disruptions in lipogenesis during metabolic stress. These findings highlight the complex relationship between metabolic stress, BCS restoration, and MFG characteristics, with implications for milk fat synthesis in lactating cows.

## 1. Introduction

The quality of milk is, in part, determined by its lipid fraction. As milk fat (MF) remains a topic of ongoing debate but constitutes a significant component of the human diet, research focused on elucidating the factors that influence its composition is crucial for enhancing its overall quality [1,2,3]. Furthermore, milk fat plays a pivotal role in determining the suitability of milk for various processing applications [4,5,6]. Milk fat, as described by Mather and Keenan [7], is predominantly synthesized from substrates provided by the diet. These substrates are initially modified in the rumen and liver before being transported via the bloodstream to the mammary gland. Within the mammary gland, these substrates undergo further modification as they traverse the cytoplasm of epithelial cells, ultimately aggregating into larger structures. These structures are then released from the milk-producing cells in the form of milk fat globules (MFGs), with over 95% of the total milk fat present in this form [8]. The characteristics of MFGs are influenced by a variety of factors. Fleming et al. [9] demonstrated that both breed and individual traits of cows play a role in determining these characteristics. Additionally, numerous studies have highlighted the significant impact of dietary factors and seasonal variations on MFG properties [5,10,11,12]. The size of an MFG is also closely related to the stage of lactation, as evidenced by the work of Mesilati-Stahy and Argov-Argaman [13]. Research conducted by Huppertz et al. [14] further substantiates the notion that the size of MFGs in bovine milk undergoes considerable changes, suggesting the involvement of multiple factors, including the intensity of lactogenesis.

One of the primary factors contributing to the considerable variation in the size of milk fat globules (MFGs) may be the mechanism of lipogenesis, which is highly energy-intensive during lactogenesis. Additionally, the characteristics of milk fat are likely to be closely associated with the systemic accumulation of fatty acids, as lactogenesis itself initiates a negative energy balance, leading to the spontaneous activation of energy reserves stored in adipose tissue [15]. During NEB, one of the most significant physiological stresses on the body is the marked increase in the systemic concentration of fatty acids [16]. This elevation in fatty acid levels leads to excessive infiltration of the liver and impairment of its metabolic functions, including glycogenesis [17]. The release of long-chain fatty acids from adipocytes, triggered by NEB, is a critical process in this regard [18,19,20]. According to Mann et al. [21], these fatty acids, particularly C16 and C18, can serve as useful biomarkers of the metabolic state of energy balance as they enter the bloodstream in their unaltered form and can subsequently reach the mammary gland.

Consequently, a systemic excess of fatty acids, originating from spontaneous lipolysis in adipocytes, may facilitate their increased uptake by mammary gland cells and incorporation into MFGs. Studies by Lopez [22], Mesilati-Stahy et al. [23], and Walter et al. [24] support this hypothesis, demonstrating correlations between the fatty acid profile of milk fat and MFG size. Furthermore, research by Couvreur et al. [25] and Ménard et al. [26] has shown that the size of MFGs is also correlated with the overall milk fat content. This relationship is of particular importance, as the milk fat content may increase in cows experiencing significant loss of adipose tissue during the course of lactogenesis. However, not all studies report a correlation between NEB and blood fatty acid levels in cows [27], suggesting that the ability of cows to efficiently utilize excess fatty acids for milk fat synthesis during intensively developing lactogenesis may be a crucial adaptive mechanism in response to NEB.

Although the effects of negative energy balance in dairy cows are relatively well documented, its impact on the characteristics of milk fat globules remains inadequately explored. The primary aim of this study is to investigate the correlations between selected biomarkers of NEB—including fatty acids (FAs) released from adipocytes, fluctuations in body condition score, and concentrations of β-hydroxybutyrate—and specific MFG characteristics, such as diameter (DMFG) and the distribution (PMFG%) of individual MFG size classes. This research is especially relevant given the continuous advancements in the production potential of dairy cows, particularly within the Holstein-Friesian breed, which is predominantly used for high-yield milk production. Understanding the relationship between metabolic disturbances, such as NEB, and alterations in milk fat composition is critical for optimizing strategies aimed at improving milk production and quality. Moreover, the findings of this study may provide valuable insights for refining management practices designed to mitigate the negative consequences of NEB on dairy cow productivity and health.

Hypotheses:

**Hypothesis** **1:** *Elevated concentrations of circulating fatty acids, resulting from adipose tissue lipolysis during NEB, will exhibit a significant correlation with changes in the size and distribution of milk fat globules, particularly the diameter (DMFG) and the proportions of larger MFG size classes (PMFG%).*

**Hypothesis** **2:** *The restoration of body condition score in lactating cows will be inversely correlated with the systemic release of free fatty acids, thereby influencing milk fat globule size and mitigating the adverse effects of NEB on milk fat composition.*

**Hypothesis** **3:** *Increased β-hydroxybutyrate levels, as a marker of NEB, will correlate with alterations in milk fat globule size, potentially leading to an increase in smaller MFGs and a reduction in the overall diameter, reflecting a compromised lipogenic pathway during metabolic stress.*

## 2. Results

Table 1 presents the results obtained for energy balance (EB) and selected parameters from calving to restoration of the body condition. As expected, a systematic increase in the energy deficit was observed from calving to the peak of production. The increase was greatest between SL 1 and SL 2, when the EB value decreased by 3.7 MJNEL/d (*p* ≤ 0.05). From SL 2 to SL 3, the EB value fell less rapidly (on average by 2.8 MJNEL/d; *p* ≤ 0.05). The EB values reached their lowest levels in SL 2 and 3. In SL 4, when the BCS was restored (Table 2), the EB value improved considerably; the difference was 16.6 MJNEL/d (*p* ≤ 0.05). The fastest increase in DMP in the population was noted up to SL 2 (32 days of lactation) and amounted to 9.9 L/d (*p* ≤ 0.05). After this time, until the cows reached peak milk production (SL 3: 61 days of lactation), DMP increased on average by 3.6 L/d (*p* ≤ 0.05). DMP decreased during SL 4 (about 132 days of lactation), on average by 5.1 L/d (*p* ≤ 0.05), and the scale of production was similar to SL 2.

The content of milk fat (MF) in the milk showed an increasing pattern up to SL 3. The average difference between SLs during this time was 0.15% (*p* ≤ 0.05). In the next SL, the content of MF decreased, which was confirmed by a difference of 0.17% (SL 4, *p* ≤ 0.05).

The levels of the fatty acids analyzed in the milk were shown to increase from SL 1 until SL 3 (Table 1). The greatest changes during this time were noted for C16:0, which increased on average by 2.4% (*p* ≤ 0.05); this was 9.9% higher than the value obtained in SL 1. A similar high value (10.5%) was shown for C18:2, although the difference in this case (SL 1–3) was only 0.21% (*p* ≤ 0.05). The smallest increase during the period from SL 1 to SL 3 was recorded for C18:0 (0.24% FA; *p* ≤ 0.05), while the share of C18:1 increased by 0.58% (*p* ≤ 0.05). The changes were similar in both cases, averaging 6.6% in relation to the values from SL 1. In the period SL 3–4, decreases were shown in the levels of FAs, ranging from 0.09 (C18:0) to 2.5 (C16:0) (*p* ≤ 0.05). The values were similar to those obtained in SL 1. The tendencies observed for the percentage of fat in the milk are also confirmed in the case of its relationship to the intensity of release of FAs into the blood. The correlation coefficients presented in Table 3, ranging from 0.667 to 0.801 (*p* ≤ 0.05), indicate that lipolysis had a strong effect on milk fat content.

Higher milk density was noted during SL 1–3, averaging 1.031 g/cm^3^. Milk density was lower after the cows had reached the peak of production (SL 3), on average by 0.004 (*p* ≤ 0.05). In addition, milk density was shown to be positively correlated, albeit only slightly, with FAs released to the blood during lipolysis (Table 4). The coefficients in this case ranged from 0.208 to 0.408 (*p* ≤ 0.05).

Table 2 presents the values of markers characterizing the consequences of negative energy balance. The data in the table show that the BCS of the cows already showed a marked decrease in the preparatory period. This is indicated by the difference between the preparatory period (PP) and SL 1, which amounted to 0.21 (*p* ≤ 0.05). This pattern persisted until the peak of production (SL 3). The greatest decrease in the BCS, however, was noted between SL 1 and 2 and amounted to 1.18 (*p* ≤ 0.05). The rate of development of lactogenesis was also highest at this time (Table 1). This pattern was confirmed by the negative correlation coefficient for DMP x BCS, which was −0.466 (*p* ≤ 0.05; Table 3).

From SL 3, a pattern of restoration of body condition was observed, as the BCS in SL 4 was 0.45 points higher (*p* ≤ 0.05). The average BCS, however, was still lower than that recorded in the preparatory period (PP). A strong correlation between SL (day of lactation) and the BCS is indicated by the relatively high positive correlation coefficient of 0.606 (*p* ≤ 0.05; Table 3).

The data presented in Table 2 indicate that the most significant changes in FA content in blood were observed for C16:0. The greatest differences in the proportion of this FA were noted approaching the peak of lactation, between SL 1 and 4. During this period, the proportion of C16:0 in the blood increased by an average of 1.51% (*p* ≤ 0.05). In the subsequent period (SL 3–4), a further increase in its proportion was observed, averaging 0.42% (*p* ≤ 0.05). However, this was comparable to the increase observed between PP and SL 1, where it amounted to 0.32% (*p* ≤ 0.05). A similar trend, although with a less pronounced increase, was observed for C18:0 in the blood. The proportion of this FA increased most rapidly during the development of lactogenesis, particularly between SL 1 and 3 (*p* ≤ 0.05), with an average increase of 0.63% (*p* ≤ 0.05). After the peak production (SL3), the increase in C18:0 content became less pronounced, as evidenced by a smaller difference of 0.24% (*p* ≤ 0.05). A different trend was observed for C18:1 (t-10, t-11) after the peak of lactation, where a reduction in the proportion of this FA was noted, averaging 0.34% (*p* ≤ 0.05). The smallest variability in FA proportion in the blood was observed for C18:2 (n-6), as indicated by similar differences both before SL 3 and after the peak of lactation. The data in Table 3 reveal that the effect of the day of lactation on the release of FAs from adipocytes was not great, as the correlation coefficients ranged from −0.287 to −0.361 (*p* ≤ 0.05). A much greater effect on the release of FAs was exerted by the intensity of lactogenesis. In the case of FAs × DMP, the correlation coefficients ranged from 0.403 to 0.524 (*p* ≤ 0.05; Table 3).

The data in Table 2 show the highest rate of increase in the concentration of BHBA between PP and the onset of lactogenesis (SL 1), when the increase in its concentration in the blood increased by 4.9% (*p* ≤ 0.05). Up to the peak of lactation, (SL 3), the BHBA concentration increased by 1.9% (*p* ≤ 0.05). After SL 3, the concentration of this biomarker showed a decreasing pattern. The direction of the changes was confirmed by the difference of 0.113 mmol/L (*p* ≤ 0.05).

The tendencies observed in this case are confirmed by the data in Table 3, which additionally indicate that the BHBA concentration in the blood was influenced to a greater extent by DMP than by the day of lactation. The correlation coefficients obtained for these parameters with BHBA were 0.569 and −0.284, respectively (*p* ≤ 0.05; Table 3).

The GLU concentration (Table 2) markedly decreased between PP and SL 1. The difference was 0.29 mmol L (*p* ≤ 0.05), which was 0.29%. Subsequently, in the period of developing lactogenesis, the average reduction in GLU in the blood amounted to 0.17 mmol/L (*p* ≤ 0.05), a decrease of 6.8%. In SL 2 and 3 its level was similar. As in the case of other biomarkers of NEB, an increasing pattern was observed for the GLU concentration in the period after the peak of production; in SL 4 it was increased on average by 0.11 mmol/L (*p* ≤ 0.05). The tendencies observed are confirmed by the correlation coefficients between the GLU concentration and DMP and the day of lactation (−0.319 and 0.309; *p* ≤ 0.05), presented in Table 3, but they indicate that their effect was small.

The tendencies observed for changes in DMFG in all size classes (Table 4) indicate that the diameter increased in all categories up to SL 3. However, the most pronounced change in DMFG was observed for class L. In this case, the differences in DMFG between SLs ranged from 0.64 µm (SL 1–2, *p* ≤ 0.05) to 0.21 µm (SL 3–4, *p* ≤ 0.05). In classes M and S, the differences in DMFG between SLs were less pronounced, averaging 0.235 µm (*p* ≤ 0.05). After SL 3, DMFG decreased again in all size classes. This change was slowest in class S; the difference in this case was 0.17 µm (*p* ≤ 0.05).

Analysis of the data pertaining to changes in PMFG showed that in SL 1–3 it decreased in classes S and M (Table 4). In the case of class L, the greatest change was noted in the period approaching the peak of production (SL 2–3). The difference in PMFG was 2.73% (*p* ≤ 0.05). In class M, a reduction in PMFG, on average by 1.08% (*p* ≤ 0.05), was noted during the most intensive development of lactogenesis (SL 1–2, Table 1). The reverse pattern to that observed in classes S and M was noted for class L (Table 4), although, as in class M, the change in PMFG was strongly influenced by the development of lactogenesis. This was indicated by the difference between SL 1 and 2, amounting to 2.04% (*p* ≤ 0.05). After this time, up to SL 3, the PMFG value increased more slowly. Following the peak of production (SL 3–4), a slight increase in PMFG was observed in classes M and S (on average by 0.97%; *p* ≤ 0.05) and a 1.47% decrease (*p* ≤ 0.05) in class L.

Table 5 presents the correlations linking selected production parameters and markers of negative energy balance with the characteristics of milk fat globules. The correlation coefficients (r) between SL (day of lactation) and DMFG and PMFG classes were relatively low, ranging from 0.147 to 0.192 (*p* ≤ 0.05) and from −0.136 to 0.209 (*p* ≤ 0.05), respectively. However, they confirm the tendencies observed in this case (Table 4). Much higher r values were obtained for DMP with DMFG and PMFG classes (Table 5). They confirm that the intensity of lactogenesis largely influenced the tendencies observed in this case. DMP was positively correlated with DMFG classes (0.315–0.515; *p* ≤ 0.05) and with PMFG class L (0.562; *p* ≤ 0.05). The values in PMFG classes M and S were negative: −0.288 and −0.469 (*p* ≤ 0.05). This confirms the pattern observed in the analysis of the results in Table 4.

The values shown in Table 5 confirm that the decline in the BCS (Table 2) was accompanied by an increase in diameter in all analyzed DMFG classes. The strength of the correlations was similar (from −0.246 to −0.296; *p* ≤ 0.05). In the case of the proportions of size classes, significant correlations between PMFG and the BCS were shown for classes S (−0.287; *p* ≤ 0.05) and M (−0.269; *p* ≤ 0.05), while a positive r value was shown in the case of class L (0.295; *p* ≤ 0.05). The changes in the BHBA concentration described above were also linked to the milk globule characteristics. Positive r values (0.197 to 0.506; *p* ≤ 0.05) were shown for all DMFG classes and for PMFG class L (Table 4), whereas negative values (−0.234 and −0.284; *p* ≤ 0.05) were noted in PMFG classes S and M. The highest r values for correlations between GLU and milk globule characteristics were obtained in the case of DMFG (r = from −0.211 to −0.369; *p* ≤ 0.05).

Lipolysis, analyzed on the basis of fatty acids released into the blood, significantly influenced MFG characteristics. In the case of size classes (DMFG), this was confirmed by the correlation coefficients. In general, the associations between the levels of FAs were greater in classes S and M, while in the case of PMFG, the connection with the level of FAs was less clear. A positive correlation was found only in the case of class L (0.265–0.481; *p* ≤ 0.05), while the proportion of MFGs in class S was negatively correlated with FAs (−0.278 to −0.521; *p* ≤ 0.05).

## 3. Discussion

The rate at which lactogenesis progresses up to the peak of milk production plays a crucial role in determining both the health and productivity of dairy cows [28,29]. One significant factor influencing this process is negative energy balance, which leads to the mobilization of free fatty acids from adipose tissue [16]. Our study observed similar effects, consistent with the findings of Ospina et al. [29], who demonstrated that postpartum free fatty acid concentrations exceeding 0.70 mEq/L are associated with a 1.7% increase in the risk of clinical ketosis and abomasal displacement. Although no clinical signs of ketosis were observed in our study, there was a notable increase in FA concentrations throughout lactation, particularly in C16:0, which is in agreement with the findings of Iggman et al. [30]. Additionally, the increases in C18:1 and C18:0 observed in our study align with results reported by Gross et al. [31]. These observations may be explained by the substantial storage of these fatty acids in adipocytes [30,31]. Iggman et al. [30] suggested that elevated levels of C16:0 in milk fat may result from de novo lipogenesis, which could explain why this particular fatty acid was most abundant in the blood in our study. It is important to note the association between C16:0 and insulin resistance [32,33], which has significant implications for dairy cows’ ability to cope with the physiological challenges of NEB. C16:0 is known to impair insulin sensitivity, as demonstrated by Nakamura et al. [34] in hepatocytes. This could indirectly explain the observed reduction in plasma glucose levels, which was accompanied by a more pronounced release of free fatty acids. Moreover, Bell and Bauman [35] highlighted that lactogenesis is prioritized by the body, leading to increased lipolysis as a response to the growing energy deficit. This process is crucial in dairy cows, as the correction of glucose imbalance primarily relies on hepatic gluconeogenesis [36]. Consistent with the findings of Bell and Bauman [35] and Champagne et al. [36], our study also showed that this adaptive mechanism becomes effective only after the peak of milk production. In contrast, Churakov et al. [18] reported higher mean blood glucose concentrations (3.53 mmol/L) in Holstein and Swedish Red cows. Unlike our study, they did not observe significant changes in blood glucose levels during the first four weeks of lactation, although a slight increase was noted after six weeks. In our study, this trend did not become apparent until after eight weeks of lactation. This difference may be attributed to physiological responses to changes in FA concentrations and the concurrent rise in beta-hydroxybutyrate, which serves as an indicator of liver stress. Champagne et al. [36] proposed that, in the absence of glucose precursors, cows may activate a glucose–lactate–glucose cycle [37]. This process is likely, given the substantial utilization of glucose during lactogenesis leading up to peak production. Our study also found that the rapid progression of lactogenesis significantly influenced the efficiency of FA oxidation in the liver. However, the BHBA concentrations did not suggest excessive ketogenesis, consistent with the findings of Ospina et al. [29], who reported that ketosis risk in lactating cows is associated with BHBA concentrations exceeding 12 mg/dL. A similar threshold was proposed by Dunfield et al. [38], who set the cut-off for BHBA at 1400 µmol/L. These results suggest that despite the metabolic challenges associated with NEB, the cows in our study did not show clinical ketosis, indicating effective metabolic adaptation to the energy deficit.

The formation of milk fat globules is a complex, multi-stage process that requires significant energy expenditure and can be influenced by a variety of factors [39]. One of the most important factors is the composition of the cow’s diet [40]. Schroeder et al. [41] demonstrated that supplementation with unsaturated fatty acids resulted in a 5.1% increase in milk fat content. However, in grazing cows, supplementation with unsaturated fats led to an 8.0% reduction in total milk fat, alongside an increase in the levels of long-chain fatty acids in the milk. This is consistent with the findings of Avramis et al. [42], who reported that pasture grazing promotes the formation of smaller MFGs. Their study also identified a correlation between specific FA levels and the ripening time of cheddar cheese. Wiking et al. [5] reported that MFG size is influenced by intrinsic factors such as the amount of milk fat produced and the availability of LCFAs. These conclusions are further supported by Couvreur et al. [25], who suggested that MFG size can be modulated by adjusting feeding strategies to align with the milk production potential of cows. They also proposed that MFG size may be linked to the metabolic activity of the mammary gland and individual cow characteristics. Our study suggests that during periods of spontaneous lipolysis, the mammary gland’s activity may be associated with a more intensive uptake of LCFAs. This hypothesis is supported by Smoczyński et al. [1], who showed that the core of MFGs is predominantly composed of LCFAs. However, our study’s results only partially align with the findings of Mesilati-Stahy et al. [23] and Lu et al. [43], who found that milk fractions containing smaller MFGs had a higher proportion of C18:1 and C18:2. This discrepancy may explain why the highest correlation coefficients in our study were found between specific fatty acids and the smallest MFG size class. The trends observed in our study also provide an explanation for the results of Massouras et al. [44], who, although working with sheep milk, also observed a relationship between MFG size and the concentration of LCFAs. Massouras et al. [44] noted that the levels of most short- and medium-chain fatty acids increased as lactation progressed. Additionally, they found that smaller MFG secretion increased during the later stages of lactation. The correlation coefficients between FAs and MFG characteristics in our study are partially consistent with the findings of Pan et al. [45], who reported that smaller MFGs had higher levels of C18:1 and C18:2 compared to larger MFGs. It is important to note, however, that Pan et al. [45] did not account for the effects of energy balance in their study. An energy deficit, such as that occurring during negative energy balance, may lead to the release of LCFAs from adipose tissue, which could influence the interpretation of their results. This possibility is supported by the additional associations observed in our study between fatty acid levels, lactogenesis progression, and body condition score loss, with relatively strong correlation coefficients. These findings suggest that lipolytic processes and the energy deficit associated with NEB may contribute to changes in the FA profile and size of MFGs. Barbano et al. [40] also suggested that these effects could arise from dietary modifications. Enriching the cow’s diet with specific fatty acids has been shown to increase the concentration of C18:1 in milk fat. This is primarily due to the biohydrogenation of unsaturated fatty acids, which increases the supply of stearic acid and oleic acid to the mammary gland. Therefore, dietary supplementation strategies aimed at increasing the availability of unsaturated fatty acids could influence both MFG size and the overall FA composition of milk.

The size of milk fat globules is a critical determinant of milk’s suitability for processing, particularly in cheese production. This aspect has been extensively discussed in the literature, with notable contributions from Logan et al. [46], who investigated the correlation between MFG size and the structural integrity of casein micelles. Their study highlighted that, in addition to influencing curd quality, the rheological properties of cheese are also significantly affected by MFG size. Specifically, Logan et al. [46] observed that MFGs of a size that allows them to be retained within the micro-capillaries of the casein curd can substantially enhance the viscoelastic properties of the final cheese product, particularly its elasticity. This characteristic is crucial for determining the texture and mouthfeel of the cheese, which are essential for consumer acceptance and quality standards. Similarly, Michalski et al. [4] reported that, regardless of cheese type, milk with a higher proportion of smaller MFGs results in a product with improved elasticity and reduced hardness. This is due to the larger surface area-to-volume ratio of smaller MFGs, which facilitates more efficient interaction with casein micelles, thus improving the gelation process and curd formation. These interactions significantly influence the rheological behavior of the cheese, including moisture retention, firmness, and structural integrity. The results of the present study suggest that negative energy balance may act as a critical cofactor in modulating the characteristics of milk fat. NEB, which occurs when a cow’s energy expenditure exceeds its energy intake, triggers the mobilization of fatty acids from adipose tissue, primarily to meet the energy demands during early lactation. This mobilization leads to changes in the fatty acid profile of milk fat, potentially affecting the size and composition of MFGs. As a result, the metabolic adaptations associated with NEB can significantly impact milk composition and its suitability for processing, particularly for dairy products requiring specific textural and rheological properties. Given the role of NEB in altering milk fat characteristics, efforts to enhance the production potential of dairy herds—particularly in terms of lactogenesis intensity—should incorporate strategies to mitigate the negative effects of NEB. Effective dietary management, which ensures a balance between energy intake and nutrient requirements, can prevent excessive NEB and stabilize milk fat composition. Such strategies could optimize milk quality and improve overall dairy herd performance. To gain a deeper understanding of the relationship between NEB and MFG characteristics, further research is needed. Specifically, studies should focus on the metabolic capacity of cows to manage the physiological stress induced by NEB, including the regulation of lipolysis and fatty acid mobilization from adipocytes. Understanding how these processes influence the synthesis and structure of MFGs could provide valuable insights into how NEB affects milk composition, particularly in terms of its functionality in dairy product manufacturing. Moreover, elucidating the underlying biochemical mechanisms could lead to more precise management practices that improve both cow health and milk quality for industrial processing.

## 4. Materials and Methods

### 4.1. Cows

This study was carried out on two dairy farms (F1 and F2) with 50 and 75 Holstein-Friesian cows, respectively. For the purposes of the experiment, 40 cows were selected from each farm, for a total of 80 cows. The main criteria for selecting cows for the study were similar milk production during the lactation preceding the study and the absence of post-partum complications.

The cows on each farm constituted separate feeding groups. The average lactation yield (±SD) in these groups was 9653 ± 259 kg (F1) and 9548 ± 341 kg (F2). The cows’ average lactation number was 2.9 ± 0.5 (F1) and 2.8 ± 0.4 (F2). The average contents of milk constituents (protein, lactose, and dry matter) during the study (% ± SD) were 3.51 ± 0.47, 4.73 ± 0.28, and 12.54 ± 0.22, respectively (Bentley Combi 150; Bentley Instruments, Inc., Chaska, MN, USA).

The cows were kept in barns with resting boxes with free access to feed (feed barrier) and water (automatic drinkers). Fans were used in summer to ensure thermal comfort. The cows were kept under conditions meeting the requirements of good production practice and animal welfare. The herds were under the regular supervision of a veterinarian.

### 4.2. Control of the Cows’ Diet and Body Condition

The cows’ diet was analyzed based on the content of nutrients in the total mixed ration (TMR) [47]. The cows’ average demand for nutrients was established according to nutritional standards [48] and calculated in INRAtion 4.06 software (INRA, Paris France). Nutritional requirements were calculated for cows with a body weight of 600 kg and daily milk production of 35 kg (3.3% protein and 4.1% fat).

The same feed ingredients were used in both herds (kg/day/cow): maize silage (22.7–25.5), barley (0.4–0.7), oats (0.5–0.9), wheat (0.5–0.7), triticale (0.9–1.4), rapeseed meal (0.7–1.2), soybean meal (2.1–2.4), NaCl (0.02), and chalk (0.1–0.2). The cows had unlimited access to salt licks as microelement supplements (NaCl—94.0%, Mg—0.20%, Co—0.18%, Zn—0.80%, Mn—0.83%, I—0.1%, Se—0.1%, water-insoluble compounds—4.0%). The TMR was prepared in a feed wagon and fed to the cows about every eight hours, using feed pushing in the intervals. About 14 days before calving, the cows received a preparatory diet. The diet was adjusted on the basis of the body condition score, daily yield in the intervals analyzed, and the amount of uneaten feed (dry matter intake (DMI) was monitored about every three days).

### 4.3. Calculation of Energy Balance

Energy balance (EB) was calculated from the amount of energy provided with the diet and its expenditure for milk production. Milk production was corrected using the energy value calculated on the basis of the actual content of fat, protein, and lactose in the milk (E_milk_), as described by Sjaunja et al. [49]. EB was calculated as daily energy intake (E_intake_) minus the energy used for milk production and maintenance of lactogenesis (E_milk_). E_milk_ was expressed in MJ, using a conversion factor of 4.19 J/cal [50]. E_intake_ was calculated using information on average dry matter intake during the periods analyzed. The values obtained were multiplied by the energy supplied with one kilogram of TMR (Table 6). Net energy demand was calculated on the basis of metabolic body weight (BW) as BW 0.75 × 0.08. Net energy in kilograms of milk production was calculated using the following formula: (0.0929 × % fat) + (0.0588 × % protein) + (0.0395 × % lactose) [51]. Energy balance was expressed in MJ NEL per day (EBMJNEL/d).

### 4.4. Collection of Material for Analysis

Blood was collected from the preparatory period (14 days before calving) to the restoration of body condition in the cows. During lactation, milk was collected and information on daily milk production (DMP) was gathered. Lactation was divided into the following stages (SLs): SL 1: 6–30 days of lactation, SL 2: 31–60 days of lactation, SL 3: 61–100 days of lactation, SL 4: 101 days of lactation to restoration of body condition. The material for analysis was collected on days 9 (SL 1), 32 (SL 2), 61 (SL 3), and 132 of lactation (SL 4—period of restoration of body condition). These time points were selected to capture key physiological and metabolic changes during lactation, such as the transition from early to mid-lactation and the recovery of body condition, which are critical for understanding metabolic adjustments in cows.

Milk was collected twice a day (morning and evening). The volume of the control sample was proportional to the amount of milk obtained. Milk was collected using the MilkMetr Tru-Test milk meter (New Zealand). The samples were refrigerated (+4 °C). Milk from both milking sessions was combined into a single sample. About 250 mL of milk was used for analysis.

Blood was collected from the abdominal vein, prior to feeding, into single-use tubes (MedlabProducts Ltd., Raszyn, Poland). Blood was placed in tubes with a clot activator except for the glucose test, for which tubes with sodium fluoride were used. The blood was stirred gently to avoid hemolysis. The material was placed in a portable refrigerator (+4.0 °C ± 1.0; CDF18; DanLab, Białystok, Poland). In the case of GLU, the tubes were placed on ice to maximize inhibition of glucose metabolism. The glucose concentration was determined on the day of blood collection. Plasma was separated from the remaining samples by applying 3500 × g/16 min at 4 °C; MPW-352RH; DanLab, Białystok, Poland) and stored at 75 °C ± 1.0 until analysis.

### 4.5. Markers of Negative Energy Balance and Lipolysis

The course of lipolysis was characterized on the basis of changes in body condition and the blood concentrations of selected non-esterified fatty acids (FAs; % of total fatty acids): C16:0, C18:0, C18:1 (t-10 and t-11), and C18:2 (n-6). These acids were selected because they are accumulated in the highest amounts in the adipocytes, where they are stored as a primary source of energy. As lipolysis occurs, these fatty acids are released into the bloodstream, reflecting the breakdown of fat reserves. Specifically, C16:0, C18:0, C18:1 (t-10 and t-11), and C18:2 (n-6) are key markers for studying lipid mobilization, as they play crucial roles in metabolic processes and are readily mobilized during periods of negative energy balance.

The rate of changes in the BCS was calculated based on body condition scores obtained in the SLs. The BCS was expressed using a five-point scale [52] as the average of scores assigned independently by two individuals. The first score was determined eight days before expected parturition (preparatory period). The scoring was conducted manually, and to reduce potential subjectivity, standardized assessment criteria were applied.

The intensity of the NEB was characterized as the energy balance, expressed in MJ NEL per day (EBMJNEL/d), and the concentrations of β-hydroxybutyrate and glucose in the blood.

### 4.6. Milk and Fat Characteristics

Raw milk was analyzed for the content of milk fat (MF); the content of FAs: (% of total fatty acids) C16:0, C18:0, C18:1 (t-10 and t-11), and C18:2 (n-6), density (g/cm^3^); and characteristics of milk fat globules (MFGs), i.e., their diameter (DMFG) and the proportion (PMFG%) of each size class: small (S: ≤3.0 µm), medium (M: 3.1–6.0 µm), and large (L: ≥6.1 µm).

### 4.7. Analytical Procedures

The density of the raw milk (g/cm^3^; standardized for 20 °C) was determined using a thermodensimeter (1015–1045 G/CM3; DANLAB, Białystok, Poland). The content of MF in the milk was determined by the Röse–Gottlieb method [47]. The analyses were performed in triplicate, and the average from the three replicates was calculated.

MFGs were counted and measured on microscope slides prepared from a mixture (*v*/*v*) of raw milk with glycerin (1:10). The measurements were made at room temperature. The mixture was applied to a microscope slide with a Thoma cell counting chamber (VWR International Sp. zo.o., Gliwice, Poland) and examined under an OLYMPUS BX-45 microscope (OLYMPUS EUROPA GMBH, Hamburg, Germany). Three slides were prepared from each sample, and photographs were taken of randomly selected areas. Measurements of MFGs were made on the images using Cell software (Olympus, EUROPA GMBH, Hamburg, Germany). Based on the measurements, the average values for DMFG and the number of MFGs per ml of milk were calculated. The proportion of MFGs in each class (PMFG%) was calculated in Statistica 13.0 software (Stat Soft Inc., Tulsa, OK, USA).

Milk fat was separated by centrifuging the milk at 3500 rpm; +4 °C ± 0.5/20 min (MPW-352RH; DanLab, Białystok, Poland). Residual moisture was removed by transferring the fat to filter paper and leaving it for about 12 h at +4 °C. Then, the fat was mixed and used for analysis.

The contents of FAs in the plasma were determined following their extraction using a mixture of hexane and isopropanol prepared in a 1:1 volume ratio [53]. The levels of FAs in raw milk were determined following extraction by the Röse–Gottlieb method [47]. The levels of the fatty acids (raw milk and plasma) were determined by gas chromatography/mass spectrometry (GCMS: Agilent Technologies Inc., Wilmington, DE, USA). Separation was carried out in a 100 m × 0.250 mm column (HP-88; SN:UST458414H, Agilent Technologies Inc., USA). The temperature program was as follows: injector 250 °C; furnace 95 °C (5 min), 120 °C (15 °C/min for 15 min), 210 °C (25 °C/min for 30 min), 250 (20 °C/min for 5 min). Carrier gas flow (H): 0.7 m L/min. Identification and percentages (% of total fatty acids) were based on retention times and standards (Supelco 37, No:47885-U; Sigma Aldrich, St Louis, MO, USA). Fatty acids were identified and their content was determined using Chemstation software (A09.03 Agilent Technologies Inc., Wilmington, DE, USA).

The levels of BHBA and GLU in the blood were determined using Randox kits (Randox Laboratories Ltd., Crumlin, UK). Their concentrations were determined in serum using the UV-Vis spectrophotometer (Varian Inc., Palo Alto, CA, USA). For BHBA the absorbance was measured at 340 nm and for GLU at 510 nm.

### 4.8. Statistical Analysis

Statistical analysis of the results was performed using Statistica 13.0 software (Stat Soft Inc., Tulsa, OK, USA). The results were analyzed for normality of distribution using the Kolmogorov–Smirnov test. Since the cows were from different farms, prior to the statistical analysis the effect of diet was compared (F1, F2; Table 6) using cluster analysis, taking into account the actual energy intake and DMI in the groups. The results were analyzed using a linear model (GLM) with repeated measurements for each SL. The results were presented as means (least squares method—LSM) and standard error of the mean (SEM). The significance of differences between means was determined by Duncan’s test at *p* ≤ 0.05. Due to the tendencies observed in the case of values for NEB markers, correlations were calculated using a curvilinear regression model (*p* ≤ 0.05).

## 5. Conclusions

This study provides valuable insights into the intricate interplay between negative energy balance and milk fat globule characteristics in lactating dairy cows, with an emphasis on understanding the molecular mechanisms underlying the observed changes. The hypotheses tested in this investigation were largely supported by the data, revealing a significant correlation between elevated circulating fatty acid concentrations—resulting from adipose tissue lipolysis during NEB—and alterations in the size and distribution of MFGs. Specifically, systemic fatty acid release was found to influence the diameter and the proportion of larger MFG size classes (PMFG%), indicating a metabolic shift that directly affects milk fat composition.

The restoration of the body condition score in cows undergoing lactogenesis was shown to be inversely correlated with the systemic release of free fatty acids, suggesting that improvements in the BCS may mitigate the adverse effects of NEB on milk fat synthesis by regulating lipolytic activity. Furthermore, the study confirmed that β-hydroxybutyrate levels, a biomarker for NEB, were associated with changes in MFG morphology, particularly a reduction in the overall diameter and an increase in the proportion of smaller MFGs, indicating potential disturbances in lipogenesis under metabolic stress.

These findings underscore the complex metabolic regulation of milk fat synthesis and highlight the pivotal role of NEB in influencing milk fat globule characteristics. The results emphasize the importance of strategies aimed at reducing NEB and improving body condition, with potential applications in optimizing milk production and quality in dairy cows, particularly in high-yielding breeds such as Holstein-Friesians.

## Figures and Tables

**Table 1:** Daily milk production and content of constituents in the milk of Holstein-Friesian cows during stages of lactation (SL).

Parameter	Stage of Lactation (SL)				SEM
	1	2	3	4	
Number of samples (*n*)	76	69	67	62	
EB (MJNEL/d)	−8.2 ^c^	−11.9 ^b^	−14.7 ^a^	+1.9 ^d^	3.5
DMP (L/day)	22.4 ^c^	32.3 ^b^	35.9 ^a^	30.8 ^b^	0.44
Milk fat (%)	4.33 ^b^	4.52 ^a^	4.42 ^ab^	4.25 ^c^	0.03
FAs (%):					
C16:0	24.26 ^c^	25.28 ^b^	26.66 ^a^	24.16 ^c^	0.10
C18:0	12.11 ^b^	11.97 ^a^	12.35 ^a^	12.05 ^ab^	0.04
C18:1 (t-10, t-11)	0.87 ^c^	1.02 ^ab^	1.08 ^a^	0.98 ^b^	0.02
C18:2 (n-6)	2.00 ^b^	2.06 ^b^	2.21 ^a^	2.02 ^b^	0.03
Density (g/cm^3^)	1.030 ^b^	1.032 ^a^	1.031 ^ab^	1.027 ^c^	0.001

a, b, c, d ≤ 0.05; EB—energy balance; DMP—daily milk production; FAs—fatty acids.

**Table 2:** Changes in the body condition and blood biomarkers of the consequences of NEB in Holstein-Friesian cows during lactation.

Parameter	PP	Stage of Lactation (SL)				SEM
		1	2	3	4	
Number of samples (*n*)	78	76	69	67	62	
BCS (point)	2.74 ^a^	2.53 ^b^	2.21 ^c^	2.04 ^d^	2.49 ^b^	0.09
FAs (%):						
C16:0	23.72 ^c^	26.61 ^b^	26.79 ^a^	27.21 ^a^	25.13 ^b^	0.14
C18:0	9.26 ^d^	10.22 ^c^	10.86 ^ab^	11.10 ^a^	10.59 ^b^	0.06
C18:1 (t-10, t-11)	2.98 ^c^	3.39 ^b^	3.38 ^b^	3.72 ^a^	3.32 ^b^	0.11
C18:2 (n-6)	2.03 ^d^	2.13 ^c^	2.20 ^ab^	2.24 ^a^	2.17 ^b^	0.01
BHBA (mmol/L)	0.87 ^c^	1.06 ^a^	1.08 ^a^	1.08 ^a^	0.96 ^b^	0.01
GLU (mmol/L)	2.97 ^a^	2.68 ^b^	2.55 ^c^	2.51 ^c^	2.64 ^b^	0.02

a–d ≤ 0.05; BCS—body condition score; FAs—fatty acids; BHBA—β-hydroxybutyrate; GLU—glucose.

**Table 3:** Correlations (r) linking selected production parameters and milk characteristics with markers of negative energy balance.

Parameter	DMP (L/day)	MF (%)	Milk Density (g/cm^3^)	Day of Lactation
BCS (point)	−0.466 *	−0.242 *	−0.149 *	0.606 *
BHBA (mmol/L)	0.569 *	0.631 *	0.235 *	−0.284 *
GLU (mmol/L)	−0.319 *	−0.336 *	0.176 *	0.309 *
FAs (%)				
C16:0	0.403 *	0.801 *	0.408 *	−0.287 *
C18:0	0.522 *	0.542 *	0.397 *	−0.344 *
C18:1(t-10, t-11)	0.524 *	0.678 *	0.388 *	−0.361 *
C18:2 (n-6)	0.522 *	0.592 *	0.296 *	−0.351 *

*—*p* ≤ 0.05; DMP—daily milk production; MF—milk fat; FAs—fatty acids; BCS—body condition score; BHBA—β-hydroxybutyrate; GLU—glucose.

**Table 4:** Characteristics of fat globules in milk fat separated from milk during stages of lactation.

Parameter	Stage of Lactation (SL)				SEM
	1	2	3	4	
Number of samples (*n*)	76	69	67	62	
DMFG class (µm)					
Small (S)	1.15 ^c^	1.39 ^b^	1.54 ^a^	1.37 ^b^	0.04
Medium (M)	4.56 ^b^	4.74 ^a^	4.83 ^a^	4.55 ^b^	0.05
Large (L)	9.05 ^c^	9.67 ^a^	9.78 ^a^	9.52 ^b^	0.04
PMFG class (%)					
Small (S)	71.04 ^a^	70.65 ^b^	67.92 ^c^	68.92 ^c^	0.18
Medium (M)	20.11 ^a^	18.49 ^b^	19.57 ^b^	19.97 ^a^	0.06
Large (L)	8.82 ^c^	10.86 ^b^	12.59 ^a^	11.12 ^b^	0.20

a, b, c ≤ 0.05MFGs—milk fat globules; DMFG—diameter of MFGs; PMFG—proportion of MFGs.

**Table 5:** Correlations (r) linking selected production parameters and markers of negative energy balance (blood biomarkers) with characteristics of milk fat globules (MFG).

Parameter	DMFG Class (µm)			PMFG Class (%)		
	S	M	L	S	M	L
DMP (L/d)	0.442 *	0.315 *	0.515 *	−0.469 *	−0.288 *	0.562 *
Day of lactation	0.147 *	0.192 *	0.152 *	−0.136 *	−0.143 *	0.209 *
BCS (point)	−0.261 *	−0.296 *	−0.246	−0.287 *	−0.269 *	0.295 *
BHBA (mmol/L)	0.317 *	0.197 *	0.506 *	−0.284 *	−0.234 *	0.391 *
GLU (mmol/L)	−0.363 *	−0.349 *	−0.211 *	0.147 *	0.126 *	−0.108 *
FAs (%)						
C16:0	0.378 *	0.268 *	0.264 *	−0.278 *	0.142 *	0.265 *
C18:0	0.587 *	0.624 *	0.308 *	−0.507 *	ns	0.427 *
C18:1(t-10, t-11)	0.534 *	0.371 *	0.305 *	−0.361 *	ns	0.398 *
C18:2 (n-6)	0.681 *	0.592 *	0.341 *	−0.521 *	ns	0.481 *

*—*p* ≤ 0.05; ns—no significant; DMP—daily milk production; FAs—fatty acids; BCS—body condition score; BHBA—β-hydroxybutyrate; GLU—glucose; DMFG—diameter of MFGs; PMFG—proportion of MFGs; MFG size class: S—small, M—medium; L—large.

**Table 6:** Nutritional value and nutrient balance of the diet (farms 1–2) during the analyzed lactation period in Holstein-Friesian cows.

Parameter	F1	F2
Number of cows	40	40
Dry matter	42.4	41.6
Protein	17.5	16.9
Fiber	18.9	19.2
Fat	2.3	2.5
Ash	8.2	8.1
Starch	22.2	22.5
Acid detergent fiber—ADF (%)	22.3	21.9
Neutral detergent fiber—NDF (%)	38.5	39.2
Physically effective NDF—peNDF (%)	29.8	30.9
UFL	21.4	21.6
PDIN (g)	2508	2497
PDIE (g)	2193	2189
Energy (MJ NEL):		
Requirement	152.8	149.9
Intake	154.2	151.6
Balance	+1.4	+1.7
Dry matter intake (kg/day)	23.2	22.9

## Data Availability

All data generated or analyzed during the study are included within the article.
